# Effect of elaidic acid on ABCA1 expression in raw 264.7 cells. Is it through PPAR-gamma?

**DOI:** 10.17179/excli2018-1605

**Published:** 2018-08-28

**Authors:** Hossein Montakhab-Yeganeh, Hossein Babaahmadi-Rezaei, Mahmood Doosti

**Affiliations:** 1 Tehran University of Medical Sciences, Department of Clinical Biochemistry, Tehran, Iran; 2 Department of Clinical Biochemistry, Ahvaz Jundishapur University of Medical Sciences, Ahvaz, Iran

**Keywords:** trans fatty acid, atherosclerosis, ABCA1, gene expression

## Abstract

In recent years, Trans Fatty Acids have shown a strong correlation with cardiovascular disease. However, the mechanisms explaining their atherogenicity are still unclear. ABCA1, which is involved in the reverse cholesterol transport pathway, has been considered as a new therapeutic target for cardiovascular disease. *In vitro* studies of the effects of PPAR-γ on lipid homeostasis in macrophage cells suggested a role for PPAR-γ in the regulation of ABCA1-dependent cholesterol efflux to apoA-I pathway. Thus, in this study we examined the effect of elaidic acid (EA) as the most abundant TFA on expression of ABCA1 and PPAR-γ in RAW 264.7 mouse macrophage cell line. Accordingly, after determining appropriate concentrations of EA using MTT, RAW 264.7 cells were treated with different concentrations of EA, and at the end, gene expression was assayed by Real-Time PCR. Our results shown that the expression of ABCA1 decreased in the treated group in comparison with the control group by 1.7, 2.3, and 5.1 fold, after 12 h treatment for 0.5, 1, and 2 mM EA concentration respectively. In addition, after 24 h treatment with EA, the rate of decreasing ABCA1 expression was 2.1, 2.6, 5.7 fold, respectively (P < 0.01). However, EA had no significant effect on PPAR-γ mRNA expression. Therefore, it could be concluded that the atherogenic effect of EA may be mediated by reducing ABCA1 expression in RAW 264.7 cells; however, this reduction has not mediated through altering PPAR-γ expression.

## Introduction

Trans fatty acids (TFA) with at least one double bond in the trans configuration, are formed during the partial hydrogenation of commercially processing vegetable oils (Bhardwaj et al., 2011[2]). Growing evidence indicates that consumption of Trans Fatty Acids (TFA) increase the risk of cardiovascular diseases (Iqbal, 2014[9]) including Coronary Heart Disease (CHD), Myocardial Infarction (MI), and Thrombotic Stroke (TS) (Virtanen et al., 2014[32]). The effects of TFA consumption on risk factors included adverse lipid effects, for example reduction in High-Density Lipoprotein cholesterol (HDL-C), raising in level of Low-density Lipoprotein cholesterol (LDL-C), and increasing tumor necrosis factor-α activity, interleukin-6, C-reactive protein that all of them have proinflammatory potential (Estadella et al., 2013[4]). Therefore, it is important to establish how TFA affect plasma lipid metabolism, and consequently result in atherosclerosis. In this view, recently several transcriptional factors have been found as regulators of the expression of a set of genes involved in lipid metabolism (Shimobayashi and Hall, 2014[28]). Among them, peroxisome proliferator-activated receptors (PPARs), belonging to the superfamily of nuclear receptors (NRs), have been identified to play a critical role in the transcriptional control of the genes encoding proteins involved in the lipid metabolism (Thakur and Srivastava, 2017[31]; Wahli and Michalik, 2012[33]). By the time, three isoforms have been identified including: PPAR-α, PPAR-β, and PPAR-γ (Kota et al., 2005[13]). To date many evidence has been demonstrated the functional significance of PPAR-γ in atherosclerosis disorder. Notably, expression of PPAR-γ has been demonstrated in atherosclerotic plaques formation and many findings suggest that PPAR-γ may be involved in the early stages of human atherosclerosis (Moore et al., 2001[20]; Sueyoshi et al., 2010[29]). However, by this time the effect of TFA on the expression of PPAR-γ has not been investigated, thus survey of this influence of TFA appears to be an important matter. On the other hand lipidation of apolipoprotein A-I (apoA-I) by ATP-binding cassette transporter A1 (ABCA1) in reverse cholesterol transport (RCT) that generate plasma high density lipoprotein (HDL) plays an important role in cholesterol homeostasis (Rothblat and Phillips, 2010[27]). With respect to RCT pathway, PPAR-γ may contribute. Nevertheless, by this time it has not been demonstrated. Therefore, it seems investigating the role of PPAR-γ in this pathway is needed, because it could elucidate the mechanism of this receptor in this important pathway. Moreover, by means of it, new points for treatment or prevention from atherosclerosis and related disorders will be created. RCT is considered as a critical step in the cardio-protective reverse cholesterol transport pathway, whereby excess cholesterol is transported from the arterial wall, especially from macrophages, and consequently delivered to the liver for excretion into bile (Rosenson et al., 2012[26]). This pathway is known the most important mechanism by which HDL can remove Free Cholesterol (FC) from peripheral cells (Yin et al., 2010[36]). Accordingly, the current study was conducted to elucidate the effect of EA (the most important isomer of TFA) on cholesterol efflux from macrophage doing by alteration in expression of ABCA1 and whether this is relevant to the PPAR-γ expression, which might have accounted for the conflicting results observed previously by others (Fournier et al., 2012[6]; Park et al., 2014[23]).

## Materials and Methods

### Materials

RAW 264.7 cells were attained from Pasteur Institute of Iran; Cell culture reagents including RPMI, HEPES, bovine serum albumin (BSA), penicillin and streptomycin media supplement, DMSO, elaidic acid (EA) and fetal bovine serum (FBS) were obtained from Sigma-Aldrich (Sigma, Deisenhofen, Germany) and Gibco (Life Technologies GmbH, Karlsruhe, Germany). SYBR Green Real-Time PCR Master Mix, Kits and reagents for RNA extraction (RNeasy plus Mini Kit), reverse transcription and primers (ABCA1, PPAR-γ, β-Actin) were purchased from Qiagen (Hilden, Germany). 

### Toxicology assay

The viability of cells treated by elaidic acid (EA) was qualified spectrophotometrically by the MTT assay. Accordingly cell viability based on MTT (3-[4,5-dimethylthiazol-2-yl]-2,5-diphenyl tetrazolium bromide) reduction by viable cells, was used to create an LC_50_ (the concentration of a chemical that kills 50 % of cells). Macrophages were cultured with EA for 24 h in 96-well microtitre plates. After 3 h incubation with MTT (0.5 mg/ml) at 37 °C, formazan crystals were dissolved in DMSO and after 10 minute of incubation, the absorbance was measured at 540 nm. 

### Cell culture

RAW 264.7 macrophages were cultured in RPMI 1640 medium containing 10 % heat inactivated FBS, 1 % penicillin/streptomycin, and Gln and incubated at 37 °C in 5 % CO_2_. Then 2 ×10^6^ cells were plated in 6 well plates, and cultured in the presence of EA-BSA (1 % BSA, in 3 different concentrations of elaidic acid including: 0.5, 1, and 2 mM) and any of these treatment were performed in 2 times of period (for 12 h and 24 h).

### RNA extraction and cDNA synthesis

The cell culture medium was removed and centrifuged to obtained cells. Total RNA was extracted by RNeasy mini kit (Qiagen, USA) according to the manufacturers' instructions. RNA purity was quantified spectrophotometrically and RNA integrity was assessed with agarose gel electrophoresis. Then RNA was reverse transcripted in a 10 µl reaction containing 1 µl Reverse Transcriptase, 4 µl RT buffer, and RT primer Mix (according to quantiTect Reverse Transcription Kit Qiagen, USA instructions) per reaction. The reaction was performed for 15 min at 42 °C, followed by heating at 95 °C for 3 min. 

### Real time PCR assay

The expression of ABCA1 and PPAR-γ was assayed by Real-time PCR on a Rotor-Gene 6000 system to determine the expression of ABCA1 in macrophages after exposition to media enriched or not (standard medium or control group) with EA. One µl cDNA with predesigned primers for ABCA1, β-actin and PPAR-γ genes (QuantiTect Primer Assay, Qiagen, USA) was amplified in separate tubes using SYBR premix Ex Taq (TaKaRa BIO INC, Japan). The condition of Real time was: 95 °C for 15 sec, followed by 40 cycles of 95 °C for 5 sec, and 60 °C for 25 sec. A standard curve was plotted by performing real-time PCR on serial dilutions of prepared cDNA and changes of gene expression were relatively quantified using ΔΔCT method. All tests were performed in duplicate and all experiments were repeated three times.

### Statistical analysis

The data represented as mean ± standard deviation, is from one experiment, which was performed at least two times. All statistical analyses were done using Prism 6.01 (Graph-pad Software Inc., USA) and Microsoft Excel 2016. One-way ANOVA and when required a Student's t test was used for the comparison between different groups, and a p < 0.05 was considered as significant. 

## Results

### Effect of Elaidic acid on the viability of RAW 264.7 macrophage cells

**Figure 1:**
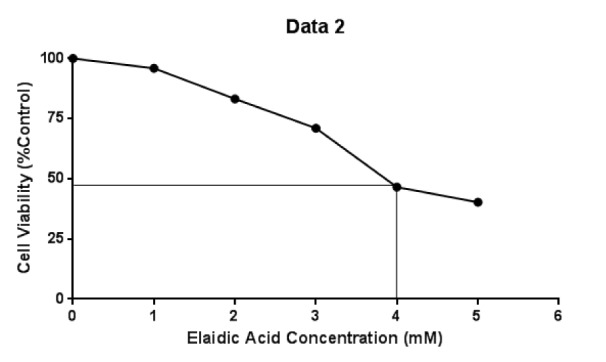
Effect of EA concentration on RAW 264.7 macrophage cell viability. RAW 264.7 macrophage cells were treated by 1, 2, 3, 4 and 5 mM of EA for 24 h. Viability of macrophages was determined by the MTT assay, and the results were expressed as the percentage of control cells treated with medium only. Values show mean ± SE of three independent experiments.

The effect of different concentrations of EA on the viability of THP-1 cells is shown in Figure 1(Fig. 1). The approximate 50 % inhibitory concentrations (IC_50_) of EA on RAW 264.7 macrophage cells at 24 h treatment were shown to be 4 mM. 

### Effect of SAC on ABCA1 and PPAR-γ mRNA expression in RAW 264.7 macrophage cells 

**Figure 2:**
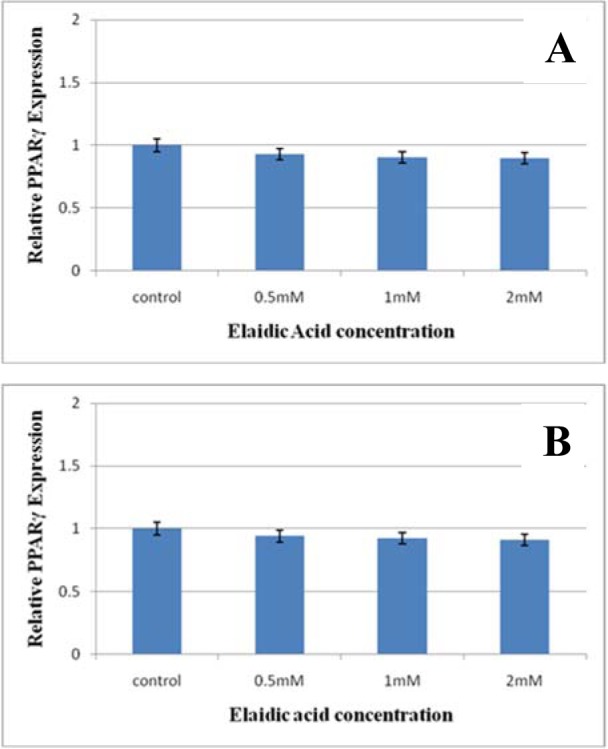
The effect of Elaidic acid treatment on the PPAR-γ expression in macrophage RAW 264.7 cells. The cells were incubated two times of periods (A: 12 h, B: 24 h). The concentration of Elaidic acid was 0.5, 1, and 2 mM, in 3 different repeats. In any times of treatment and all concentrations of EA, the PPAR-γ mRNA expression did not show any statistically significant changes (P < 0.06).

**Figure 3:**
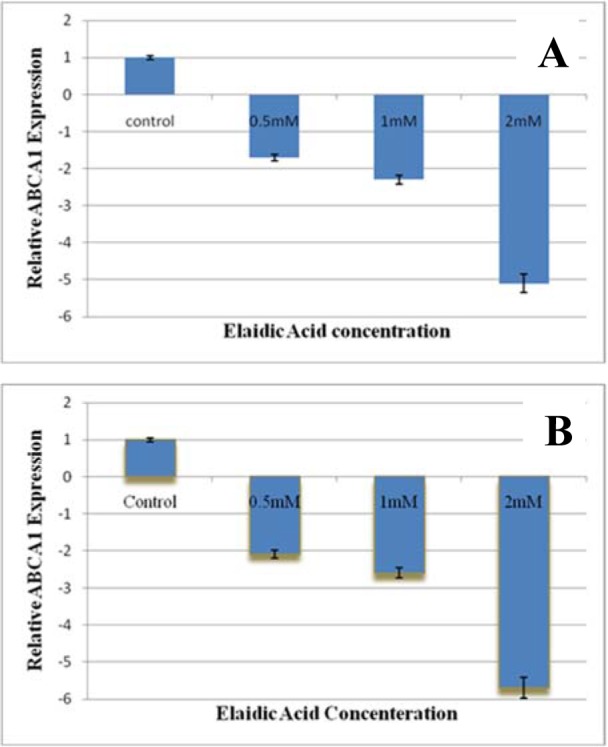
The effect of Elaidic acid treatment on the ABCA1 expression in macrophage RAW 264.7 cells. The cells were incubated two times of periods (A: 12 h, B: 24 h). The concentration of Elaidic acid was 0.5, 1, and 2 mM, in 3 different repeats. However, unlike PPAR-γ the level of gene expression of ABCA1 significantly decreased respectively by 1.7, 2.3, and 5.1 fold, after 12 h treatment. In addition, gene expression of ABCA1 after 24 h treatment was deceased 2.1, 2.6, and 5.7 fold respectively (compared with control group) (P < 0.01). In all conditions of treatment, the experiments were done three times.

ABCA1 mRNA levels were measured in RAW 264.7 macrophages exposed to media enriched or not (Standard medium) with EA semiquantitative Real-Time RT-PCR assay. The effect of 0.5, 1, 2 mM concentration of EA on the expression of ABCA1 and PPAR-γ genes are presented in Figure 2(Fig. 2). The mRNA levels of ABCA1 decreased in the treated group in comparison with the control group 1.7, 2.3, and 5.1 fold after 12 h treatment by 0.5, 1, and 2 mM concentration of EA, respectively. In addition, the rate of decreasing after 24 h treatment was 2.1, 2.6, 5.7 fold respectively (in the 0.5, 1, and 2mM EA concentration compared with control group) (P < 0.01). However, as illustrated in Figure 3(Fig. 3), EA could not cause any alteration in the gene expression of PPAR-γ at statistical value. 

## Discussion

Our major focus in the present study was to investigate whether EA could affect the gene expression of ABCA1, and PPAR-γ in macrophage cells. We found no major differences over time in the expression of PPAR-γ mRNA in the RAW 264.7 mouse macrophage cells treated by EA, but the expression of ABCA1 decreased in this cell line. Therefore, it can be concluded that the expression of ABCA1 was not mediated by PPAR-γ. However, previous studies have shown that the gene expression of PPAR-γ decreased in macrophages treated by EA (Rao and Lokesh, 2017[24]; Yegane et al., 2012[19]). Some studies reported that TFA increased the production of inflammatory markers such as CRP, TNFα, and IL6 (Bendsen et al., 2011[1]; Longhi et al., 2017[15]). It seems the lipid composition of macrophage membrane plays a substantial role in macrophage function (Remmerie and Scott, 2018[25]). Accordingly insertion of TFA into membrane lipids makes it more rigid as compared to saturated or cis unsaturated fatty acids (Magarkar et al., 2014[16]). Hence, the presence of TFA in membranes alters activation and functions of macrophages. So according to current results it can be concluded that EA impairs the cholesterol efflux mediated with ABCA1 in macrophages through decreasing the fluidity and rigidity of the membrane. The expression of PPAR-γ, however, did not show any significant change after treatment by EA. Altogether, the possible explanation for the observed results including the adverse effect of TFA could be mediated by another transcription factor such as LXR, HNF4a, FXR or other isoform of PPARs. In one study for example it was reported that the expression of ABCA1 is stimulated by PPARα in foam cell formation in a liver X receptor-dependent manner, promoting apoAI-mediated cholesterol efflux (Nakaya et al., 2011[21]). In addition, it is likely that TFA reduces the particle size of LDL-c in a dose-dependent fashion (Mauger et al., 2003[17]). Another study has demonstrated that TFA increase the cellular accumulation and secretion of free cholesterol and cholesterol esters from hepatocytes (Dashti et al., 2002[3]). According to this study and in combination with our result, the incorporation of TFA in the membrane of macrophages is more growing up. 

Several recent clinical studies have reported that dietary trans fatty acids negatively affect the plasma lipoprotein profile in humans (Takeuchi and Sugano, 2017[30]; Yang et al., 2017[35]). The association of TFA consumption with coronary heart disease risk and atherogenicity have been shown in numerous epidemiological studies and meta-analysis of well-designed controlled trials, but their relevant effects according to the mechanisms underlying their atherogenicity are still controversial (Kisioglu and Nergiz-Unal, 2017[12]; Nestel 2014[22]). Reverse cholesterol transport (RCT) is believed to be a key cardio-protective mechanism emphasized *in vivo* mouse models appreciating the relationships between effluxes of cholesterol from macrophages (He et al., 2018[8]). The availability of a partially hydrogenated soybean oil preparation, in which elaidic acid (EA) predominated (Jakobsen et al., 2007[11]; Larque et al., 2001[14]) allowed us to use EA rather than other fatty acids. In the literature concerning the potential mechanisms through which TFA increase the risk of CHD, the effects of TFA on plasma lipoprotein profiles and lipid metabolism have been extremely studied (Fakhar et al., 2015[5]; Gates et al., 2017[7]). Our aim was to study the *in vitro* effects of EA treatment on the expression of two most important possible genes, which play a critical role in lipid metabolism. For this purpose, we exposed cells to EA, and then studied the consequences of this treatment on ABCA1 and PPAR-γ mRNA expressions. Here, the EA treatment of RAW 264.7 cells induced an increase in ABCA1 expression without affecting PPAR-γ. This result suggests that the atherogenicity of EA in RAW 264.7 mouse macrophages could be modestly attributed to the ABCA1 down expression by no effect on the expression of PPAR-γ mRNA. Considering the high relevance of foam cells formation in atherosclerotic lesions development, these results might be relevant due to the significance of ABCA1 in RCT compared to the other mechanisms (Wang et al., 2007[34]). The exact mechanism of the alteration of ABCA1 functionality by TFA had not been explored but we can raise the sensible hypothesis that there is no relevance with PPAR-γ mRNA expression. Compared with this study a few investigations have shown that TFA could impair endothelial functions by altering vasodilatation (Iwata et al., 2011[10]). Furthermore, on the inflammatory aspect we have previously demonstrated that EA had no effects on expression of TNF-α gene as one of important cytokine involving in systemic inflammation, although other cytokines such as IL-6 could be considered (Montakhab-Yeganeh et al., 2017[18]). Taken together, these results suggest that the reduction of ABCA1 mRNA expression caused by EA treatment is not attributed to PPR-γ pathway and decreasing in ABCA1 expression is progressed in another mechanism.

## Acknowledgement

We would like to acknowledge Dr. Parisa Shaabani (Semnan University of Medical Sciences) for English editing of the manuscript. This study was funded by the grant from the Tehran University of Medical Sciences (Grant ID. TUMS-2152) to Mahmood Doosti. The funder had no role in study design and interpretation of the work.

## Conflict of interest

All authors declare that they have no conflict of interest.
